# Stewardship to tackle global phosphorus inefficiency: The case of Europe

**DOI:** 10.1007/s13280-014-0614-8

**Published:** 2015-02-15

**Authors:** Paul J. A. Withers, Kimo C. van Dijk, Tina-Simone S. Neset, Thomas Nesme, Oene Oenema, Gitte H. Rubæk, Oscar F. Schoumans, Bert Smit, Sylvain Pellerin

**Affiliations:** 1 Bangor University, Bangor, Gwynedd LL57 2UW UK; 2 Wageningen University, P.O. Box 47, 6700 AA Wageningen, The Netherlands; 3 Linköping University, 58183 Linköping, Sweden; 4 Bordeaux Sciences Agro, CS 40201, 33175 Gradignan Cedex, France; 5 Alterra Wageningen UR, P.O. Box 47, 6700 AA Wageningen, The Netherlands; 6 Department of Agroecology, Aarhus University, Blichers Allé 20, P.O. Box 50, 8830 Tjele, Denmark; 7 Plant Research International, University and Research Centre, Wageningen, The Netherlands; 8 INRA, UMR 1391 ISPA, CS 20032, 33882 Villenaved’Ornon, France

**Keywords:** Phosphorus, Europe, Inefficiency, Eutrophication, Human health, Resources, Sustainability

## Abstract

The inefficient use of phosphorus (P) in the food chain is a threat to the global aquatic environment and the health and well-being of citizens, and it is depleting an essential finite natural resource critical for future food security and ecosystem function. We outline a strategic framework of 5R stewardship (Re-align P inputs, Reduce P losses, Recycle P in bioresources, Recover P in wastes, and Redefine P in food systems) to help identify and deliver a range of integrated, cost-effective, and feasible technological innovations to improve P use efficiency in society and reduce Europe’s dependence on P imports. Their combined adoption facilitated by interactive policies, co-operation between upstream and downstream stakeholders (researchers, investors, producers, distributors, and consumers), and more harmonized approaches to P accounting would maximize the resource and environmental benefits and help deliver a more competitive, circular, and sustainable European economy. The case of Europe provides a blueprint for global P stewardship.

## Introduction

In a recent introductory essay, Schipper (2014) suggested that the time has now come for some form of phosphorus (P) stewardship on the grounds that P is far too important a resource to be wasted on such a large scale by society. Phosphorus is a ubiquitous but hidden element in our world and essential for cellular function, reproduction (DNA, RNA), and human development (Westheimer 1987; Richardson 2009). It is an important nutrient input in our crop and animal production systems, and therefore has a high economic value. A future shortage of P would threaten global food security, bioenergy production, and alter ecosystem structure and function due to the resulting stoichiometric imbalance in the stocks and utilization of nitrogen (N) and carbon (C) (Neset and Cordell 2012; Peñuelas et al. 2013). Food and energy security are also highly dependent on clean water which becomes degraded when the P status is high. The management of this finite P resource is therefore of critical importance for the development of circular economies that deliver sustainable growth with zero waste, minimum use of primary raw materials, and least environmental damage (EC 2014b).

Since the 1950s, society has become dependent on the processing of phosphate rock (PR) to produce a range of concentrated, soluble inorganic P compounds used in fertilizers, feed supplements, food additives, and detergents. While there is both on-going and some ‘alarmist’ debate on exactly how much global PR reserves we have left (Edixhoven et al. 2013; Ulrich et al. 2013), there is no disputing the gross inefficiency with which society uses these P compounds. The environmental damage (eutrophication) from unused and lost P is widespread and costly affecting ecosystem diversity, human health, well-being, and prosperity (Smil 2000; Dodds et al. 2009; Smith and Schindler 2009). There are also emerging concerns over the links between high P diets, high blood serum P, and a range of human health problems including calcium homeostasis, kidney function, cardiovascular disease, aging, and cancer (Calvo and Park 1996; Ellam and Chico 2012; Gonzales-Parra et al. 2012; Schroff 2013). This P inefficiency, and the resulting environmental and human health problems, will only become worse as a growing urbanizing global population demands more food, bioenergy, and clean water, and changes to our climate will exacerbate the eutrophication of our precious water resources. It is time society adopted more sustainable P management to help mend this broken biogeochemical cycle (Elser and Bennett 2011).

Europe has a number of issues to resolve concerning P management, and in many ways, P can be viewed as a test case for other non-renewable resources on which Europe depends. It is now becoming increasingly recognized that such resources ‘*underpin the functioning of the European and global economy and our quality of life*’ and that ‘*continuing the current [unsustainable] patterns of resource use is not an option*’ (EC 2011). Europe has very little PR reserves and is almost totally dependent on P imports, mostly as fertilizer (Ott and Rechberger 2012). This makes Europe vulnerable to future P scarcity as regional food security, and bioenergy demands would be compromised if P imports became restricted, or their cost sharply increased (Cordell and Neset 2014). The European Commission has now recognized this potential future scarcity by placing PR on its list of critical raw materials (EC 2014a). As in other continents, the use of P across Europe is very unbalanced; there are farms and regions where the use of too little P is compromising agricultural output, while there are other more intensive farms and regions where the overuse of P is continually building up (legacy) P levels in soils with increased long-term eutrophication risk (Csatho and Radimszky 2009; Rubaek et al. 2013; Tóth et al. 2014). Europe’s inland and coastal waters are heavily eutrophied due to inputs of P in urban and rural wastewater discharges and in agricultural and urban runoff (EEA 2012; Carstensen et al. 2014). Phosphorus fertilizers also contain harmful metals, such as cadmium (Cd), from the impurities in the parent rock, although the risk of soil Cd accumulation and leaching across Europe is less now than it used to be (Six and Smolders 2014).

There are therefore pressing economic, environmental, human health, and resource justifications for increasing the sustainability of P use across Europe and indeed globally. Europe has a particular trilemma: it has high P demands because many regions are densely populated and have intensive agriculture and high energy and water requirements, it has extensive degradation of its aquatic ecosystems from past intensification of P use, and now its food security maybe compromised because it has virtually no PR reserves. Resolving this trilemma is the P sustainability challenge facing Europe, but how is it best addressed when there is no regulation over P use in many EU countries (Amery and Schoumans 2014)? In this article, we discuss the inefficiencies of P use across Europe and outline five key strategies (Re-align P inputs to meet only essential requirements, Reduce P losses to water, Recycle P in bioresources more effectively, Recover P in wastes, and Redefine P in food systems) that will deliver key policies, innovations, and measures to improve Europe’s P efficiency, and reduce its P imports with potential benefits to food, energy and water security, human health, global P resource management, environment, and biodiversity. We suggest these 5R strategies could act as a blueprint for global P stewardship and sustainability.

## Phosphorus use inefficiency in the European Union

**Fig. 1:**
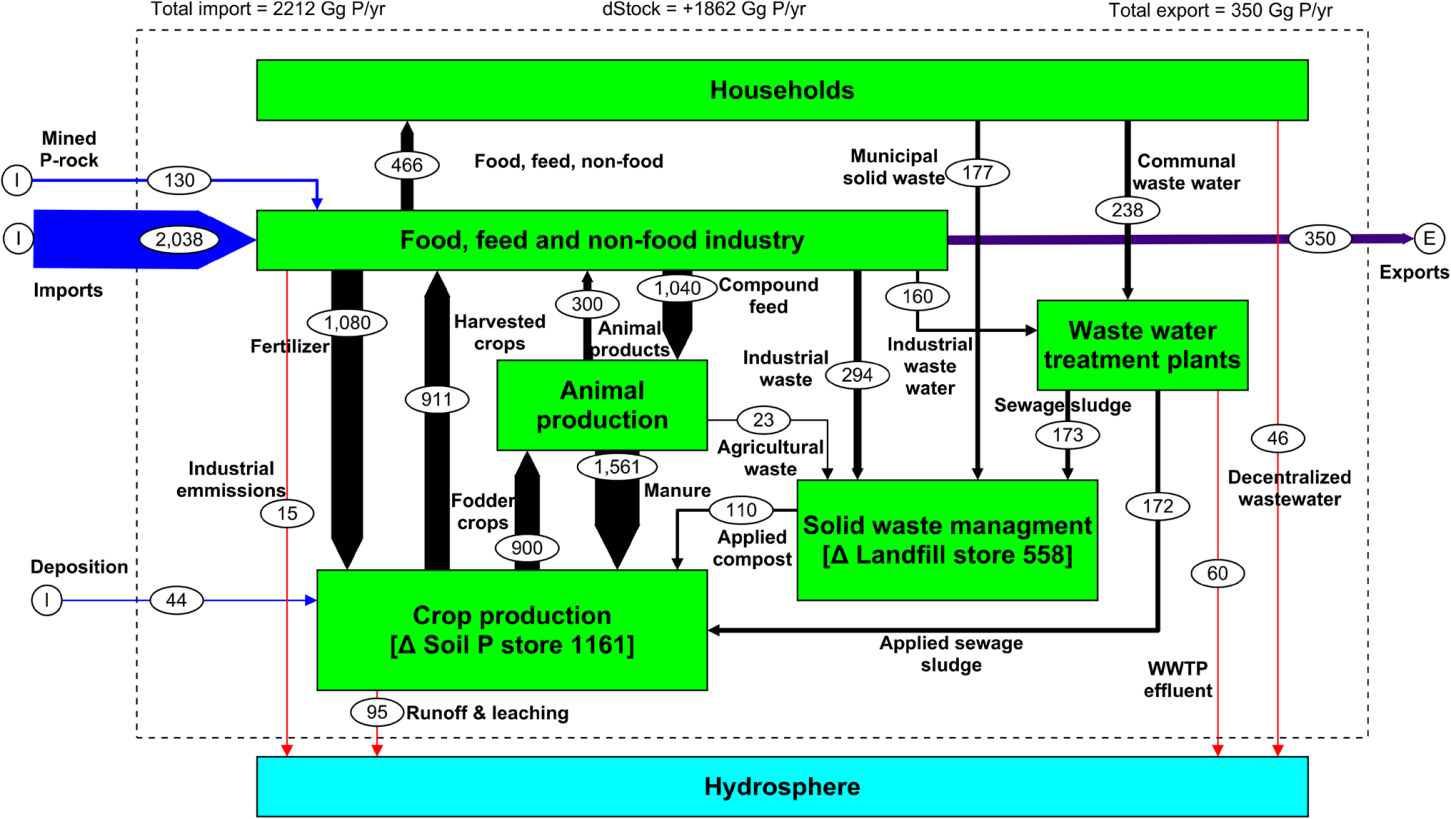
Phosphorus flows in the food production–consumption chain of the European Union (EU15) for 2006–2008. Flows are indicated by *arrows*, and pools and stocks are indicated by *boxes*. The size of flows, stocks, and pools are presented in Gg P yr^−1^. The size of the *arrows* indicates the relative size of the flow; imports are in *blue*, exports are in *purple*, losses are in *red*, sectors are in *green*, and the hydrosphere is in *light blue* (adapted from Ott and Rechberger 2012)

A P mass balance for the former EU15 countries based on average data for the years 2006–2008 using a material flow analysis (MFA) was recently reported by Ott and Rechberger (2012); the key sectors, stocks, and annual P flows in the balance are summarized in Fig. 1. Data in the figure are presented as Gg P (not P_2_O_5_) yr^−1^, but, for summary here, are given in Tg (1000 Gg) and sometimes also expressed on a per capita basis. Net balance of P (total inputs minus total exports) is estimated at 1.9 Tg P yr^−1^ (4.7 kg P per capita yr^−1^), of which over 80 % are net imports of PR-derived products for use in agriculture, the food industry, and in detergents. The food, feed, and non-food industry sectors are the main distributors of P within the cycle including 1.1 Tg P yr^−1^ in fertilizers for crop production, 1.0 Tg P yr^−1^ in protein-rich feed for livestock production, and 0.5 Tg P yr^−1^ in food and non-food products for households. Agriculture is the main user of P, and internal P flows in agricultural systems are high; for example, manure P flows are greater than that of fertilizer (Fig. 1). Overall, agriculture is about 50 % P efficient, but crop production systems are much more efficient (60 %) than livestock production systems (14 %). Unused P in agriculture largely accumulates in the soil (1.2 Tg P yr^−1^, 2.9 kg P per capita yr^−1^ or 8.6 kg P ha^−1^). Most of the P consumed in households (60 %) leaves as wastewater to be treated at wastewater treatment plants (WWTP), or as solid (e.g., food) waste to be composted, incinerated or taken to landfill. In total, WWTP receive about 0.4 Tg P yr^−1^ (1.0 kg P per capita yr^−1^), and about 0.6 Tg P yr^−1^ of solid waste is stored (lost) to landfill (Fig. 1). Losses of P to water from WWTP, industry, agricultural land, and individual households total about 0.2 Tg P yr^−1^ (0.55 kg P per capita yr^−1^).

**Fig. 2:**
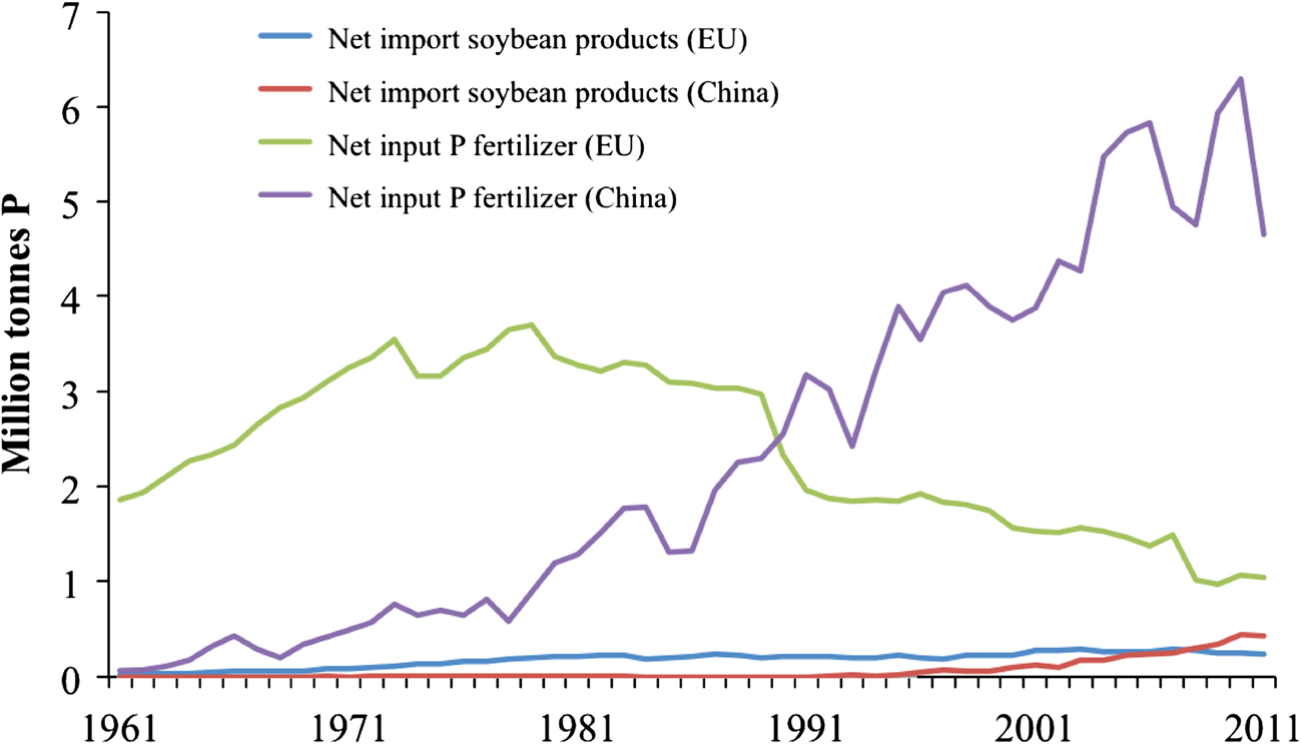
The use of phosphorus (P) fertilizers and the import of P via soybean products in EU-27 between 1961 and 2011. For comparison, results for China have been included (*Source* FAOSTAT 2014)

This first European P budget has highlighted a number of key indicators and trends of P use efficiency in Europe. These indicators and trends are supported by the results of a detailed P balance for the EU27 countries for 2005 [Van Dijk (unpublished results); some preliminary results are given in Schoumans et al. 2015], and the results of MFA undertaken in a number of countries within and outside the EU and summarized by Chowdhury et al. (2014). Firstly, the food system of the EU can be seen to be highly dependent on the import of PR-derived products, which include fertilizers (1.6 Tg P yr^−1^), animal feed (0.25 Tg P yr^−1^), food (0.1 Tg P yr^−1^), and non-food products (0.1 Tg P yr^−1^). Only an estimated 0.13 Tg P yr^−1^ enters the P cycle from small PR deposits in Finland. In sharp contrast to China, the imports of P via fertilizers in the EU have steadily decreased from about 1980 (Fig. 2), largely due to tighter profitability margins and better informed decision making on farms. However, imports of P via animal feed (especially soybean) in the EU have increased in line with greater livestock numbers. Secondly, the efficiency with which imported P is used by consumers is low; only 25 % of the net P used in the EU15 countries reaches households for human consumption (Ott and Rechberger 2012). Thirdly, most of the unused P is steadily accumulating in EU soils, with increased long-term risk of further P runoff and leaching losses to water bodies. Fourthly, there is very limited recovery and recycling of wastewater and solid waste P back to land; only about 0.3 Tg P yr^−1^ representing 16 % of net P usage is recycled to land mainly as compost and sludge. Fifthly, total losses of P to water and to landfill are substantial (0.8 Tg P yr^−1^) and account for 42 % of the net annual usage. Assuming inorganic manufactured P costs of €2 per kg P, these losses have a potential value of over 1.5 × 10^9 ^euros.

The EU P balance constructed by Ott and Rechberger (2012) also highlighted the large degree of uncertainty associated with some estimates, most notably in the amounts of P in waste recycled to land, and of losses to the hydrosphere due to the lack of and/or difficulty in accurately monitoring these flows. Differences between individual countries in inflows and outflows, the mean accumulation of P in agricultural soils, and in the recycling of wastes can be expected to be large due to differences in fertilizer use, livestock density, and government regulations (Chowdhury et al. 2014). For example, the recycling of communal sewage sludge depends on the wide variation in contents of nutrients and heavy metals they contain and the risks to human health related to these pollutants accumulating in agricultural soils (Milieu 2009). In addition to country-wide estimates, there also appears to be large variation in P cycling between regions and between sectors, and these variations along with those across years are still insufficiently understood (Senthilkumar et al. 2012; Chowdhury et al. 2014).

## Key innovations toward a more resource-efficient Europe

**Fig. 3:**
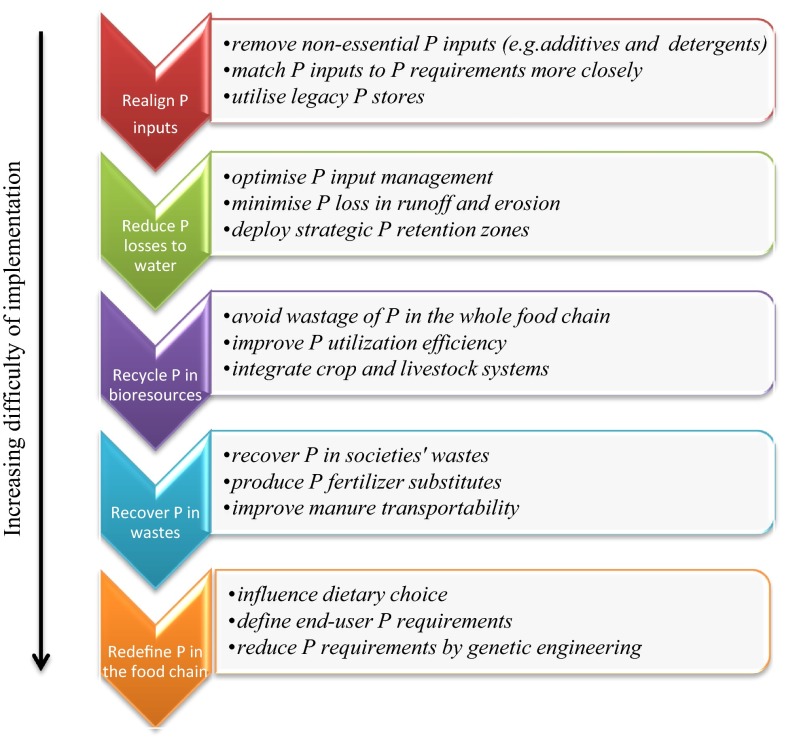
The 5R strategies to reduce Europe’s dependency on phosphate rock-derived P in the likely order of increasing difficulty of implementation

Reducing Europe’s high dependency on P imports requires innovative interventions to improve P use efficiency along the whole food chain. The 1st European Sustainable Phosphorus Conference held in Brussels in 2013 (http://www.phosphorusplatform.org/) sets three key goals: use less, recycle more, and co-operate. Following on from the outputs of two Scientific European Phosphorus Workshops (http://www.wageningenur.nl/sepw2013), we outline here 5-key R strategies in order of increasing difficulty to deliver these goals and innovations and make Europe more P sustainable (Fig. 3). This framework could be delivered by interactive policies that facilitate the combined adoption and integration of the 5R strategies to provide multiple benefits (i.e., win–win solutions). For example, increasing the digestibility of P in livestock feed may not only re-align inputs by enabling lower inorganic feed P imports but also increase the manure N:P ratio allowing better utilization on the land. Similarly, exploitation of legacy soil P stores will not only help reduce fertilizer inputs, but will also gradually reduce diffuse P emissions to water and reduce Cd inputs to soils. Other examples of potential synergies are given in Neset and Cordell (2012). Koppelaar and Weikard (2013) estimated that combined adoption of loss reduction measures (especially from agriculture), and recovery and recycling options could provide a global reduction potential of 13 Tg P yr^−1^ (over 50 %), although some options were far too costly relative to the price of PR production. The main levers, practical measures, expected progress and remaining technical constraints, and potential bottlenecks for each R strategy and some potential innovations are outlined below and summarized in Table 1.

**Table 1:** Some examples of the instruments and addressees, practical measures, and potential bottlenecks to achieve each 5R strategy

R strategy objectives	Instruments and addressees	Practical regional/farm measures	Potential bottlenecks
Re-align P inputs to match actual P requirements	Legislation to restrict overuse of P in agriculture [government, farmers’ organizations, feed companies]	Establish industry–farmer agreements on lowering the mineral P supplementation in animal feed and increase its digestibility	Limited knowledge on optimizing production methods with low P inputs (e.g., improving prediction of soil P supply)
	Improve tools and guidance to encourage better nutrient management [extension services, consultancy firms]	Mine soils with a high P content and improve the P status of soils with a low or insufficient P status	Farmer implementation of precision farming principles and practice
	Ban unnecessary P products and additives (e.g., detergents, food/feed additives) [government, industry]	Improve precision farming technologies to increase P efficiency in both livestock and cropping systems	Suitable and economic alternatives to non-essential P products
			Enforcement/control of any P input restrictions
Reduce P losses to water to minimize eutrophication risk	Define and facilitate a catchment-based approach to reduce P loads from point and diffuse sources with well defined targets [all catchment stakeholders]	Develop more accurate methods to quantify point and diffuse source contributions to eutrophication	More evidence to link agricultural P mitigation measures to ecological impacts
	Develop markets for the provision of ecosystem services whereby the beneficiaries pay land managers for their provision (e.g., upstream thinking) [research, consultancy firms]	Increase awareness of water quality issues from upstream rural land use and farm yards (e.g., septic tanks), and from urban areas	Poor uptake of measures due to lack of farmer engagement
		Select, implement and monitor a set of targeted measures to reduce P losses	Conflicts between stakeholders; for example between improving water quality and agricultural productivity
Recycle P in bioresources more effectively to substitute inorganic fertilizer consumption	Ease legislation to encourage wider use of society’s bioresources on farms and by industry [governments, farmers’ organizations, conservation agencies]	Integrate livestock and cropping systems at regional scale and fully exploit fertilizer substitution potential at field scale	Market prices for bioresources regulated by manure surplus rather than on agronomical value
	Establish regional agreements to facilitate more uniform distribution of livestock manure to arable farms [governments, farmers’ organizations]	Improve quality and value of recycled materials through better sourcing and treatment (e.g., manure treatment/separation)	Limited knowledge to support full field-scale substitution of various bioresources for fertilizers
		Refine feed formulations to increase manure N:P ratios and their uniform redistribution	Acceptability by society and the food industry of human waste derived products; for example in relation to food safety
Recover P in society’s wastes, by-products and residues for re-use	Increase societal dependence on a circular economy with recycling targets (e.g., tax on primary P imports, zero waste) [governments, industry]	Implement new technologies to recover P from society’s wastes	Time lag in taking promising recovery options/technologies to the market
	Subsidize investments in P recovery technology in collaboration with industry [governments, industry]	Improve P accounting methods to maximize opportunity for recovery in different parts of the food chain	Current technologies too uneconomic for adoption and/or reluctance to reflect the real price of primary P production by including externalities
	New business models and financing mechanisms to foster innovations to the market [industry, consultancy firms]	Minimize waste production to limit the need to recover P	Backlash on business models if subsidies are removed
Re-design P use in society with a focus on food systems	Mainstream sustainable P use into European and national legislation [governments, consumer organizations]	Increase public awareness of how dietary choice influences P demands and possible health risks of high P diets (the health and sustainability challenge)	Limited knowledge to confirm links between high blood serum P and increased human health risks
	Specify P dietary requirements and prioritize essential demands [industry, research, consumer organizations]	Lower the P contents of foods by reducing their P requirements through plant breeding and food processing	Reluctance of the public to change food habits; e.g., the focus is now on calories and proteins but not nutrients or their sustainable use
	New urban/rural spatial planning models for circular economies [governments, urban planners, consumer organizations]	Plan urban areas to maximize P recycling opportunities	Timelag in developing foods with inherently lower P contents Integrating P sustainability into urban planning

### Re-align P inputs to match requirements

The amounts of inorganic P currently added to soil, livestock rations, and food products are often in excess of actual requirements. This practice has evolved to provide an insurance element against the unpredictability of shortfalls in P supply to crops and livestock. It is questionable whether this insurance-based philosophy for P management is justified when unused (legacy) P continues to accumulate in the soil, sediments and wastes posing a threat to aquatic ecosystems and human health (Sharpley et al. 2013; Withers et al. 2014c). Conversely, there are some areas of Europe with impoverished soils where agricultural productivity is still limited by a lack of crop available P (Tóth et al. 2014). Addressing these imbalances requires a re-alignment of inorganic fertilizer P inputs to more closely match crop and animal P requirements after taking maximum account of the P supply from the soil, and from recycled manures. Clearly, any economic constraints on P use need to be taken into account, but such a re-alignment strategy would improve P efficiency, close the gap between the lowest yielding and highest yielding producers, and help minimize the environmental impacts of fertilizer overuse. Mueller et al. (2012) estimated that addressing the imbalance of P inputs to rice, maize, and wheat globally would save 38 % of P fertilizer use without reducing crop yields. Removing the insurance element in current P use means that P accounting and management will need to become more precise: for example, through decisions on the right source, rate, time, and place of P application (Bruulsema et al. 2009). Research must demonstrate how greater precision and lowering of P inputs can be achieved without loss in production, livestock fertility, or human health.

Innovations in fertilizer types (i.e., smart fertilizers), more targeted methods of application (e.g., seed dressings, placement and foliar P) and increasing adoption of precision farming technology (e.g., variable rate applicators), have potential to lower P inputs by improving fertilizer P recovery by crops (Simpson et al. 2011; McLaughlin et al. 2012; van der Velde et al. 2013). Similarly, dietary P intakes to livestock could be reduced by 20–30 % without affecting livestock performance and lead to lower surpluses and significantly less P to recycle with overall efficiency gains (Maguire et al. 2005; Ferris et al. 2010; Kebreab et al. 2012). However, such re-alignment of P in livestock diets does not necessarily mean cheaper rations because of the difficulty of sourcing ingredients for a balanced feed. Using a simple two-compartment soil P availability model, Sattari et al. (2012) predicted that the fertilizer demand for Europe up to 2050 could be cut by nearly 50 % without limiting crop yields if residual soil P (legacy P) was taken account of. Understanding regional variation in legacy P is therefore key to increasing P use efficiency in Europe (e.g., Rubaek et al. 2013).

While P is required for healthy crops and livestock, some P inputs into the food system, or in national P budgets, are not essential for life. Reijnders (2014) defines “essential uses” as uses of P in the economy for which no substitute exists or for which substitutes exist but are more of an environmental burden. Phosphorus-containing additives are widely used in human foods (e.g., as a preservative), but they are not needed to meet dietary P intake requirements (see section below). Similarly, polyphosphates used in laundry and dishwashing detergents make a substantial contribution to P loads entering Wastewater Treatment Plants (WWTP) (van Drecht et al. 2009), but are not essential and are now being gradually removed. Phosphorus is increasingly being used by water companies to reduce lead (Pb) mobilized by plumbosolvency in old water piping to comply with increasingly stringent EU regulations for Pb (e.g., Hayes et al. 2008). However, economic alternatives to P to resolve this issue are now on the market, and this P input may also be unnecessary (Comber et al. 2013). The concept of re-aligning P inputs to what is actually necessary therefore applies across both rural and urban P cycles.

### Reduce P losses to water

Agricultural intensification and urbanization during the twentieth century have greatly increased losses of P to inland and coastal waters (Smil 2000): for the EU15, annual losses amount to 0.2 Tg P yr^−1^ and are dominated by wastewater discharges and erosion/leaching from agricultural land (Fig. 1). Nutrient input reduction is a key strategy for eutrophication control (Smith and Schindler 2009), and reductions in bioavailable P loads from WWTP have been particularly successful in improving water quality and reducing the incidence of harmful algae in eutrophic ecosystems (Heisler et al. 2008; EEA 2012). However, centralized WWTP still discharge at least 0.06 Tg of P into EU15 surface waters, and further controls over P inputs to and discharges from WWTP will still be necessary to achieve the required reductions in P loads to EU coastal waters (Grizzetti et al. 2012). Wastewater discharges from rural septic tank systems could also be significant (0.05 Gg P yr^−1^, Ott and Rechberger 2012) and may have more eutrophication impact than previously thought requiring action at the household level (Withers et al. 2014a). Europe has now placed limits on the amounts of P in consumer laundry, and more recently dishwashing detergents to help reduce P loadings to WWTP (EU 2012). Technologies already exist to improve P stripping from wastewater effluent prior to discharge (for example, by biological nutrient removal and dosing with Fe/Al and Mg), and these provide an opportunity to recycle the resulting P-rich biosolids back onto suitable land areas (see section below).

Agriculture is also a major contributor of P loadings to surface waters in European catchments and globally (EEA 2012; OECD 2012). However, the beneficial impacts of mitigating P loss from agriculture are less easy to demonstrate and quantify because of their diffuse, dynamic, and unpredictable nature (Kronvang et al. 2005; Maguire et al. 2009). Losses of P in leaching and runoff across the EU15 add up to ca. 0.1 Tg P yr^−1^ (Fig. 1), but with large uncertainty. A multitude of measures have been implemented, or can be potentially deployed, to reduce P transfers from agricultural land to surface waters, including soil conservation measures to reduce soil erosion, careful management of P inputs to reduce direct losses after application, and strategic placement of P retention zones to help prevent P delivery (Kröger et al. 2013; Schoumans et al. 2014). However, there is still a general lack of understanding of (i) how widespread these options need to be implemented across catchments, (ii) which options will achieve maximum ecological gain (e.g., dissolved v particulate P control), (iii) how best to identify and manage the critical hydrologically active areas that generate the majority of the P load, and (iv) how to resolve conflicts between improving water quality and agricultural productivity and profitability (Withers et al. 2014b). Innovations to encourage more pro-active stakeholder engagement including more catchment-based approaches and payments to land managers for delivery of ecosystem (environmental) services may help resolve these issues (e.g., McGonigle et al. 2012). Of particular concern is the legacy P in soils, sediments, and groundwater that is an endemic and long-term source of P inputs to surface waters via runoff, and is delaying the restoration of good ecological quality in many surface waters (Sharpley et al. 2013). This legacy P will take many decades to reduce, and further agricultural intensification and climate change are expected to increase diffuse P losses if actions are not taken sooner rather than later (Macleod et al. 2012; Schoumans et al. 2015).

### Recycle P in bioresources more effectively

There is a long tradition of returning crop residues, animal manures, bone meal, and wastewater biosolids to the field to increase crop production by improving soil chemical and physical properties and to utilize the available nutrients they contain. A range of other bioresources including anaerobic digestates, composts, and industrial by-products are being made increasingly available for application to land to reduce the need for landfill and reduce wastage (e.g., Parfitt et al. 2010). Livestock manures represent by far the largest source of recyclable P in Europe (1.6 Tg, Fig. 1), although relatively little account is taken of their nutrient value in modern cropping systems. Over-application of manures is therefore a major problem in highly stocked areas. As inorganic P fertilizers become more expensive, the value of manures and other bioresources can be expected to increase, but research to support their full substitution for fertilizers is surprisingly lacking. Variability in the crop availability of P in different bioresources (e.g., Fe-treated biosolids, O’Connor et al. 2004) led Oenema et al. (2012) to conclude that while they were valuable in building up soil fertility, they should not be used as a substitute for fertilizers where soil P supply is critical.

Other constraints on the full utilization of Europe’s bioresources include the costs of transporting manure to suitable land areas due to the geographical segregation of arable and livestock farms, and for biosolids, the distances from urban centers, public perception of possible health hazards, and concerns over environmental contamination. Not all land is suitable to receive manures due to landscape factors (e.g., slope), existing soil contamination, or regulatory controls (Nicholson et al. 2012). Bioresources contain pathogens, metals, nanoparticles, and persistent organic chemicals which may harm soil or human health (Arthurson 2008; Clarke and Smith 2011). Manures are a major source of nitrate leaching to groundwater and ammonia emissions to the atmosphere (Petersen et al. 2007), and an unfavorable N to P ratio in many bioresources results in an over-application of P relative to crop requirement. This leads to a low efficiency of manure P utilization, accumulation of P in soils, and increased eutrophication risk (Shober and Sims 2003; Peñuelas et al. 2013). Rules and regulations exist in Europe to minimize these constraints through sanitation treatment, codes of practice to prevent contamination of crops that enter the human food chain, and guidelines on storage and application practices to reduce atmospheric emissions. However, the large volumes of manure P generated in relation to the available land area in some regions remains a major barrier to sustainable recycling (Bateman et al. 2011), especially if the EU introduces regulatory controls over organic P loadings to land to reduce eutrophication risk. Restricting manure application rates will improve P efficiency but may lead to the dispersal of livestock systems and affect farmer livelihoods if technology cannot improve manure transportability to crop-producing areas (Gaigné et al. 2011).

There is clearly large scope to reduce inorganic fertilizer P inputs through better harmonizing of manure production to the available land area, exporting excess manure to other areas, with or without prior treatment, and better integration of livestock and cropping systems to help overcome geographical disconnects. The advantages for soil function and increased resilience to climate change stress (e.g., drought) of integrated production systems that utilize bioresources are being increasingly recognized (Robertson and Swinton 2005; Herrero et al. 2010). More precise manipulation of livestock diets to reduce P in manure relative to N would help reduce soil P accumulation rates: for example, the use of biorefinery to separate out the main constituents of feeds (proteins, enzymes, phosphates) to increase their digestibility and absorption (Kebreab et al. 2012). However, there is a limit to the extent innovations in recycling can cost-effectively resolve the manure transportability issue, which may require other ‘recovery-based’ solutions.

### Recover P from wastes

Much of the P in the different wastes (domestic, agricultural, and industrial) that society generates is currently not utilized for logistical, economic, contamination, or hygienic reasons. The amount of P contained in waste is large, 0.6 Tg P yr^−1^ (Fig. 1), and its recovery into transportable and crop available products is a logical solution to improving the integration of various wastes into cropping systems with large potential savings in fertilizer use (Schoumans et al. 2015). Recovery technologies must themselves be economic, efficient, and clean, and produce a contaminant-free product that is of sufficient quality to allow its use as a fertilizer substitute, or as a secondary resource for the non-food industries (Schipper et al. 2001; Ulrich et al. 2013). In this way, recovered P products can compete with rock P-derived products to ensure that P fertilizer prices remain affordable. Recovery processes that are not directly profitable are difficult to commercialize and sustain, and future policies to support recovery technologies may need to factor in the environmental externality costs of current P use (Koppelaar and Weikard 2013).

A number of alternative technologies have been investigated to recover P from manures, P-rich sludge, wastewater, and incineration ash in the form of struvite (magnesium ammonium phosphate), mono ammonium phosphate (MAP), and calcium phosphate (CaP), and have recently been described in detail by Ohtake and Okano (2015) and Schoumans et al. (2015). Struvite recovery is potentially attractive because it has proved to be a useful slow-release fertilizer (e.g., Massey et al. 2009), but production levels and use are currently low in Europe, not least because of the specific requirement for the very P-rich wastewater produced by biological nutrient removal (Le Corre et al. 2009). Where domestic and industrial wastes are incinerated, P can also be recovered as fertilizer from the incineration ash by acid or alkali digestion with high rates of P recovery and very little contamination (e.g., Tan and Lagerkvist 2011; Donatello and Cheeseman 2013). Incineration-based recovery is an attractive waste disposal method in Europe because it produces thermal or electrical energy, and therefore the costs are acceptable, and it also makes possible the recovery of other valuable elements from the waste flows such as micronutrients (e.g., Cu, Zn, Mg) (Fytili and Zabaniotou 2008; Rulkens 2008).

Phosphorus recovery from manure has received much attention in intensive livestock areas (e.g., The Netherlands and Belgium), where manure volumes are large and land areas for their application are limited. Incineration of high dry matter manures (e.g., poultry litter) is potentially attractive because it produces energy from the organic matter and a P-rich ash (i.e., greater than in biosolids) that can be recycled to land. The disadvantage is that all valuable organic materials as soil conditioner are lost together with the valuable N-compounds to the atmosphere. Using pyrolysis to preserve, the C produces a potentially useful P biochar (Wang et al. 2014), but this approach is not common in Europe and still rather expensive (Shackley et al. 2011). Separating the solid fraction of manure by drying (e.g., in combination with biogas production), sanitizing, and pelleting reduces the costs of transport but this is not economic (Schoumans et al. 2015). Other innovative techniques for manure treatment, such as wet oxidation (sub- and super critical) and wet super critical gasification, are emerging, but more attention has focused on simple techniques to recover only a part of the P in manure through precipitation of calcium phosphates and struvite. The advantages of this manure fractionation are that the ratio of N and P in manure may become more in line with the requirements of crops, and this may reduce the need for inorganic P fertilizer and/or the export of surplus manure. Furthermore, a small volume of P-rich (but poor quality) precipitate is produced, which can be used as a secondary P resource for industries.

### Redefine P in food systems

Similar to other resources, the European diet puts significant demands on the continuous input of inorganic and organic P into the food chain. Driven by a high proportion of meat and dairy products, the European diet has a significantly higher P demand than countries like India that have a more vegetarian diet (Metson et al. 2012). The average per capita dietary P intake by adults in Europe (ca. 1.55 g P day^−1^, range 1.3–2.7 g P day^−1^, Flynn et al. 2009) is double the minimum P requirement for human health (0.7–1 g P day^−1^, EFSA 2005). While further evidence is clearly needed to confirm recent research suggesting adverse effects of high P diets and blood serum levels on human health (Schnee et al. 2014), there is clearly potential to reduce the unnecessarily high P consumption rates in food across Europe. Donner (2007) calculated that a future food system scenario with no red meat would reduce the need to grow so many crops (to feed the animals) leading to a 50–60 % reduction in the use of P (and N) fertilizers. Clearly, the impacts of such a strategy on national economies, overseas trade, meat demand by developing nations, and increased land required to grow crops profitably all need to be taken into account, but the potential impact of dietary choice on nutrient use is clearly profound (Metson et al. 2012). Food wastage is also considerable; for example, Gustavsson et al. (2011) estimated that on average, 30 % of initial food production is lost between post-harvest and consumption. A reduction of this wastage would imply significant potential benefits in reducing P losses and improving overall P efficiency. Consideration of the way urban areas are planned to accommodate agricultural ecosystem services and maximize P recycling opportunities as they grow in area in the future is another food system design innovation (Cummings et al. 2014). To achieve the necessary paradigm shift in public eating habits and industry responsibilities, developing more P-efficient food chains must be seen as a health and sustainability challenge, and the environmental P impact accounted for in the same way as for the accounting of water or carbon.

As well as redefining the food system, there is potential to reduce P contents in food. For example, the ubiquitous use of P in food additives by the food industry, primarily as preservatives, may not be necessary. Estimates differ on the relative contribution these additives make to dietary P intakes with values of ca. 10 % to over 30 % reported (Calvo and Park 1996; Comber et al. 2013; EA 2013), but, as with many aspects of P use in society, this contribution needs to be clarified and maybe relatively small. Much of the P contained in crops is in the form of phytate which is poorly utilized by monogastrics and reduces the availability and absorption of certain essential micronutrients, including iron (Fe), zinc (Zn), Ca, and magnesium (Mg), in the body of animals and humans (White and Broadley 2009). Seed total and phytate P contents show large genetic variation, and could be reduced in cereals by 20–25 % without affecting plant regeneration, or human health, through genetic engineering (see Withers et al. 2014c). The food industry does not specify their P requirements in the same way they do for N (e.g., for milling wheat and malting barley), and there is therefore scope for end users to specify what their precise P requirements are. Genetic variability in the P requirements of livestock can also be expected, but dietary inputs probably have a greater impact on the P content of animal products. The combination of lowering P in crop and livestock products and finding alternatives to inorganic P additives in human foods would help lower inorganic P input, internal P flows, and subsequent P losses to the environment.

## Overcoming barriers to change

**Fig. 4:**
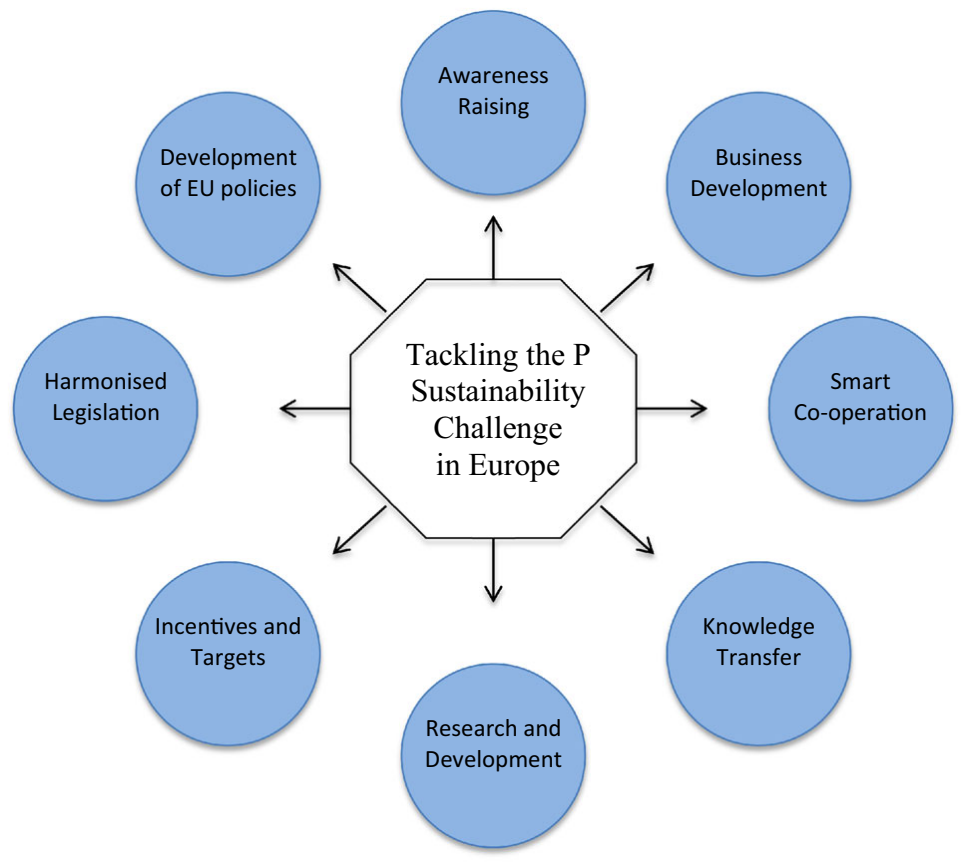
Tackling the P sustainability challenge in Europe: the eight conclusions from the 1st European Sustainable Phosphorus Conference held in Brussels in 2013 (http://www.phosphorusplatform.org/)

The placement of phosphate rock on Europe’s list of critical raw materials provides a firm platform for actions toward more sustainable P use. The 1st European Sustainable Phosphorus Conference held in 2012 identified eight ways to tackle the P sustainability challenge Europe faces (Fig. 4). A wide variety of technical innovations, business opportunities, and measures are potentially available to make Europe more P efficient (Table 1), but a robust and integrated assessment of their potential combined impact on the whole P cycle in terms of the amount of PR saved, cost, and benefits to the environment and human health has still to be carried out. Some innovations target P specifically like the development of novel P fertilizers, or engineering solutions for the recovery of P from human, livestock, and industrial waste streams. For these innovations, barriers to their adoption are expected to be mostly legislative, technological, or economical, although acceptability by society and permitting regulations may also play a role (e.g., genetically modified plants with better P uptake efficiency; struvite-based secondary fertilizers). Most innovations for better P use efficiency involve a wider process, such as improved manure recycling in agriculture, modifying food habits, or changing wastewater treatment systems. Their applicability may be limited by a wider range of barriers and constraints, due to economics, interactions, and possible antagonisms with other issues; for example, infrastructure costs, N management, or food safety. Identifying business opportunities and constraints for better P recycling and recovery requires a clear view of the whole P cycle and its drivers including food production–consumption chains, international markets, and waste management policies.

**Fig. 5:**
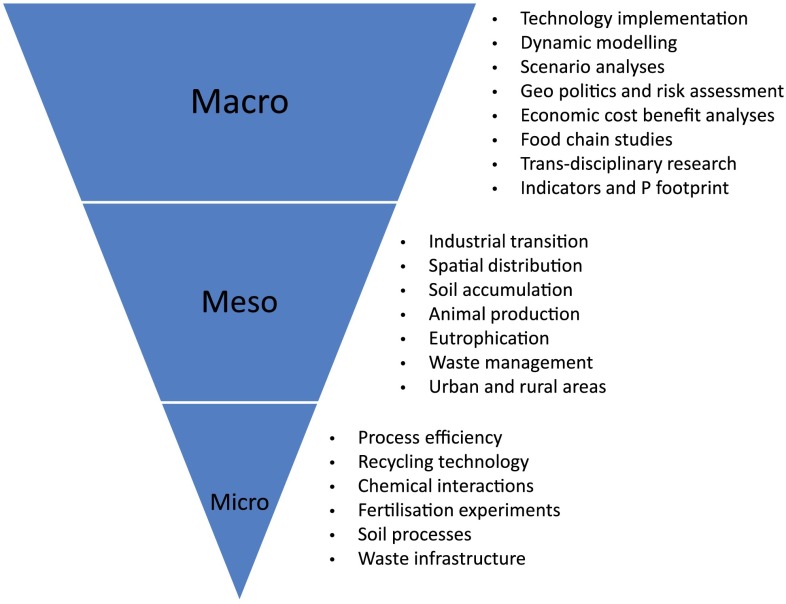
Defining a research agenda for sustainable P management through the integration of the macro-, meso-, and micro-scales of research and knowledge

Understanding the interactions and potential overall impact of current and future P use on PR reserves and our environment is a key scientific challenge. Research and development to deliver the benefits of technological innovations must be conducted across several scales (Fig. 5). At the macroscale, several research efforts have focused on P flow analyses in food chains in different countries to identify crucial points of intervention (e.g., Cordell et al. 2012; Linderholm et al. 2012; Egle et al. 2014), but there is a lack of co-ordination of notions, methods, and modeling procedures among these studies, which makes a comparison of their results and outcomes difficult (Chowdhury et al. 2014). At the meso-scale, several industries and research groups from different disciplines (social, agronomy, engineering, economics) are working on some specific segments of the P cycle, such as improving P use efficiency in plant or animal farming (Kebreab et al. 2012; Withers et al. 2014c), or P recovery from solid waste or waste water (Oenema et al. 2012; Kabbe 2013). However, their outcomes are rarely combined and integrated, and economic and policy issues are not always addressed, making a proper assessment difficult. At the micro-scale, research on basic processes underlying P efficiency still need to be translated into potential win–win solutions so that they can be demonstrated and implemented at a practical level, for example in soils and plants (Richardson 2009; Ryan et al. 2009).

Involving stakeholders from the multiple processes/sectors in the P cycle and developing appropriate business opportunities is an important challenge and key to success (Ulrich et al. 2013). There is a need to raise awareness among stakeholders about the urgency to recover and re-use P from wastes, and to utilize P more efficiently in the whole food production–consumption–waste recycling chain. For example, European consumers are major stakeholders in the P cycle but they are not always sufficiently aware of the environmental P impacts associated with their dietary preferences and handling of food (waste), or of the potential regional vulnerability of their food systems to P scarcity (Metson et al. 2012; Cordell and Neset 2014). All sectors of society need to embrace the concept of sustainability and resource use efficiency to safeguard food, energy, and water security and the preservation of a healthy and ecologically diverse environment for future generations.

## Conclusions

Europe is strongly dependent on net P imports via mineral P fertilizers, food, feed, and detergent components from foreign countries. There is large scope to reduce the dependency on especially P fertilizer imports and make Europe a more resource-efficient, competitive, sustainable, and healthy society. We have proposed in this paper a 5R stewardship framework (Re-align P inputs, Reduce P losses, Recycle P in bioresources, Recover P in wastes, and Redefine P in food systems) to help achieve these goals. The framework has been designed to be interactive to help implement the changes needed. Sound policies for sustainable P use need to be based on the best available scientific evidence, which needs to be integrated across Europe’s different geographical, political, and economic settings and involving all relevant sector and stakeholder communities. Achieving this science base and stakeholder community platform requires (i) a common and quantitative understanding of the P flows and cycling in the food production/consumption/waste management chain across Europe, (ii) an overview and evaluation of existing and emerging innovative P management options for each R strategy and across all sectors, (iii) a scenario analysis of how these possible options might be integrated across Europe for maximum resource and environmental benefits, and (iv) actions and business models for achieving a P-efficient Europe. We argue that the integrated networking of policymakers, scientists, and sector representatives across geographic regions is urgently needed to overcome the P challenge Europe faces. In this way, Europe’s P sustainability agenda can provide the blueprint for global P stewardship, and also for the sustainable use of other essential non-renewable resources (Cu, Zn, Mg), in keeping with its vision for a resource-efficient society (EC 2011). In a global context, P stewardship is of particular relevance to the emerging economies (India and China) with very high P import demands for growth. The 5R strategies are equally relevant to poor countries with limited affordable access to P and where recycling is a key part of farming practice. To what extent recovered P may be a cheaper alternative to PR products for these countries remains unclear.
